# The Gut and Parkinson's Disease—A Bidirectional Pathway

**DOI:** 10.3389/fneur.2019.00574

**Published:** 2019-06-04

**Authors:** Susanne Fonseca Santos, Hadassa Loth de Oliveira, Elizabeth Sumi Yamada, Bianca Cruz Neves, Antonio Pereira

**Affiliations:** ^1^Graduate Program in Neuroscience and Cell Biology, Institute of Biology, Federal University of Pará, Belém, Brazil; ^2^Department of Biochemistry, Institute of Chemistry, Federal University of Rio de Janeiro, Rio de Janeiro, Brazil; ^3^Department of Electrical and Biomedical Engineering, Institute of Technology, Federal University of Pará, Belém, Brazil

**Keywords:** Parkinson's disease, enteric nervous system, microbiome, neurotoxicants, probiotics

## Abstract

Humans evolved a symbiotic relationship with their gut microbiome, a complex microbial community composed of bacteria, archaea, protists, and viruses, including bacteriophages. The enteric nervous system (ENS) is a gateway for the bidirectional communication between the brain and the gut, mostly through the vagus nerve (VN). Environmental exposure plays a pivotal role in both the composition and functionality of the gut microbiome and may contribute to susceptibility to neurodegenerative disorders, such as Parkinson's disease (PD). The neuropathological hallmark of PD is the widespread appearance of alpha-synuclein aggregates in both the central and peripheral nervous systems, including the ENS. Many studies suggest that gut toxins can induce the formation of α-syn aggregates in the ENS, which may then be transmitted in a prion-like manner to the CNS through the VN. PD is strongly associated with aging and its negative effects on homeostatic mechanisms protecting from inflammation, oxidative stress, and protein malfunction. In this mini-review, we revisit some landmark discoveries in the field of Parkinson's research and focus on the gut-brain axis. In the process, we highlight evidence showing gut-associated dysbiosis and related microbial-derived components as important players and risk factors for PD. Therefore, the gut microbiome emerges as a potential target for protective measures aiming to prevent PD onset.

## Introduction

Parkinson's Disease (PD) is a common neurodegenerative disorder typically associated with the progressive loss of dopaminergic neurons located in the midbrain nucleus substantia nigra pars compacta (SNpc) (1). Although the cardinal symptoms of PD are motor impairments attributed to the depletion of the neurotransmitter dopamine in the striatum, a major target of the SNpc (2), it has been long recognized [for review, see (3)] that other non-motor symptoms, including olfactory (4–6) and gastrointestinal (GI) dysfunction (4), appear during the so-called premotor phase of the disease.

**Figure 1:**
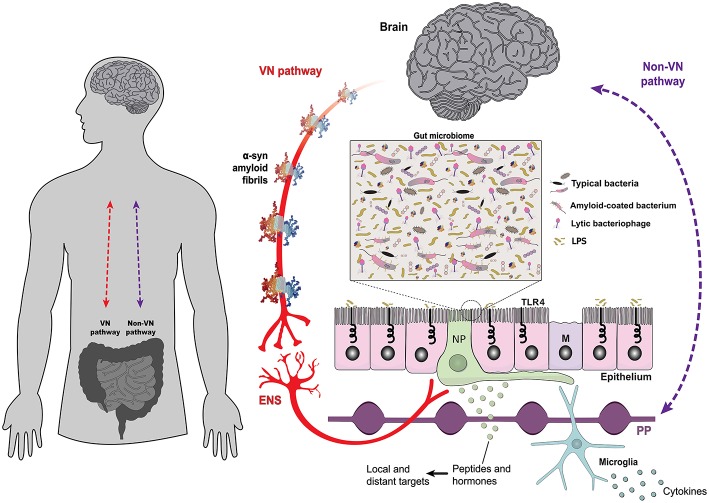
The gut epithelium is a multifunctional interface. The bidirectional interplay between the brain and the gut is mediated by neural, such as the vagus nerve (VN-gateway), and humoral pathways, such as the lymphatic tissue and the bloodstream (Non-VN gateways). A monolayer of epithelial cells separates the intestinal lumen and the complex gut microbiome from the underlying lymphoid and enteric nervous tissues. The structure of alpha-synuclein amyloid fibrils (PDB 2N0A) is based on atomic-resolution molecular data from NGL Viewer (19). Members of the gut microbiome and their extracellular compounds may trigger responses in the VN through enteroendocrine cells, which are contacted by vagus nerve terminals through specialized structures called neuropods (NP) (20). Microbial antigens can cross the gut epithelium through microfold cells, playing a central role in localized inflammatory responses [adapted from Bohórquez et al. (21)]. Toll-like receptors are microbe-sensing proteins, present in intestinal epithelial cells, mediating recognition of commensal bacteria from the harmful/inflammatory ones. ENS, enteric nervous system; M, microfold cells; NP, neuropods; PP, Peyer's patches; TLR4, Toll-like receptor 4; VN, vagus nerve.

The neuropathological hallmark of PD is the presence of cytoplasmic inclusions, called Lewy bodies (LB) or Lewy neurites (7–9), in SNpc neurons (10). LBs are composed mostly of α-synuclein (α-syn) aggregates (11–13), whose aberrant soluble oligomeric conformations are thought to mediate its toxic effects (14). Alpha-syn is an intrinsically disordered protein (IDP), which lacks a stable 3D structure under physiological conditions and is characterized by exacerbated structural plasticity and conformational adaptability (15). As other IDPs possessing amyloidogenic regions (16), α-syn can turn into a promiscuous binder leading to abnormal interactions and the development of PD (17). Tuttle et al. (18) provided a detailed 3D structure of functional α-syn fibrils (see Figure 1), using solid-state NMR spectroscopy. The study may serve as the basis for a better understanding of molecular mechanisms involved in α-syn fibril nucleation and propagation. In addition, such structural information may provide useful insights on possible interactions of α-synuclein with other proteins and small molecules and allow the emergence of new tools with potential to facilitate both the diagnosis and treatment of PD (e.g., imaging agents and therapeutic drugs).

Aggregates of α-syn fibrils are also found in neural tissue located outside the central nervous system (CNS) of PD patients, in both the autonomic and enteric nervous system (ENS), an outcome which may be associated with the non-motor symptoms of the disease [for review, see (3)]. These findings led Braak et al. (4) to propose a staging system for the progression of the disease following a specific pattern of α-syn aggregates spreading from peripheral toward more centralized locations in the brain. The triggering event would be the invasion of vulnerable neural structures such as the olfactory epithelium and the ENS, which interface directly with the external environment (5, 22), by a neurotoxicant (“neurotropic virus”) (23). While both structures (24, 25) possess immunological and physical barriers protecting them against environmental insults, these barriers steadily deteriorate with aging [for review, see (26, 27)], which is the biggest risk factor for idiopathic PD (28).

Animal studies have supported the claim that α-syn aggregates propagate in a prion-like manner [(29); for review, see (30)] via microtubule-associated transport along axons (31). In summary, the prion hypothesis of PD proposes that amyloidogenic α-syn would induce a conformational change in the endogenous protein through permissive templating, convert it into a likeness of itself (32, 33) and propagate retrogradely through the vagus nerve or the olfactory tract from the ENS or the olfactory bulb, respectively. Even though definitive proof for this prion hypothesis is still missing (30) and there is the controversial possibility that intestinal α-syn aggregates have a brain origin (34, 35), it has been shown that vagotomy is associated with a decreased risk for PD in humans (36, 37). Also, grafted neurons in PD patients develop α-syn aggregate pathology (38–40) and α-syn from PD patients can cause nigrostriatal degeneration in mice and non-human primates (41). Remarkably, exogenous α-syn fibrils, either PD patient-derived or produced in *E. coli*, were able to seed the formation of LB-like inclusions which spread from the GI tract to the brain through the vagus nerve in rats (31).

Prior to Braak's hypothesis, however, there was already strong evidence pointing to the role played by exogenous toxins in the etiology of sporadic PD. For instance, postencephalitic parkinsonism (von Economo's disease), which has an autoimmune basis caused by a viral illness (42), is associated with degeneration of the basal ganglia (43). Additionally, the discovery of parkinsonism induced by 1-methyl-4-phenyl-1,2,4,5-tetrahydropyridine (MPTP) through self-administration, in 1982 (44) brought to light a new class of xenobiotic substances that may cause PD-like symptoms by environmental contact. MPTP is a lipophilic compound which readily passes into the brain where it is converted by monoamine oxidase B (MAO-B) to 1-methyl-4-phenylpyridinium (MPP+) (45) which is taken up by dopaminergic cells and impairs mitochondria respiration by poisoning complex 1 (46). There are many heterocyclic molecules that structurally resemble MPTP and are found in the brain from both endogenous and exogenous sources, such as tetrahydroisoquinolines (TIQ) and β-carbolines (β-C). For instance, a TIQ derivative, salsolinol, which is produced by enterobacteria (47) and has been found in the urine of PD patients, may have a double-faced, dose-dependent effect on the nigrostriatal pathway as either a harmful or protective agent (48).

The evidence for the role played by toxins in inducing parkinsonism and the relative scarcity of familial cases (about 10%) (49) underscore the importance of environmental and lifestyle factors over genetic ones in the etiology of the disease (50–52). Some chronic diseases have been associated with a phenomenon called evolutionary mismatch when ancestral traits are no longer adaptive in modern contexts (53, 54). For instance, α-syn is involved with normal synaptic function by regulating, among other things, the size of presynaptic vesicles (55) and the assembly of SNARE proteins involved with the docking of synaptic vesicles to presynaptic membranes (56). However, as old age became common in humans after the early upper Paleolithic (57), the steady increase in longevity seen in modern times may have had a collateral effect on the protein homeostasis (proteostasis) network, which coordinates protein synthesis, folding, trafficking, disaggregation, and degradation (58, 59). The breakdown of proteostasis, which is a common feature of many neurodegenerative diseases (60), means that misfolded proteins may accumulate due to lack of clearance or failure to refold into their native structures (61). In the case of prion-like proteins, this may cause further protein misfolding (template effect) leading to protein aggregation and ultimately cell death (62).

## The Gut-Brain Axis and Parkinson's Disease

The gut-brain axis is mediated by intense bidirectional communication between the CNS and the ENS (63). Through the ENS, the gut microbiota influences the development and function of all divisions of the nervous system (64) and this association was established very early during the evolution of multicellular organisms. The first nervous system appeared more than 500 million years ago before the divergence of cnidarians and bilaterians, the two metazoan sister groups (65). That primitive brain had a simple structure, organized as a diffuse nerve net which controlled a restricted set of basic behaviors and was the template for the subsequent evolution of the mammalian ENS (66–68), which retained many of its basic structural characteristics, such as a network of nervous ganglia distributed in the myenteric and submucous plexuses (69). Higher vertebrates went to evolve an additional set of neural structures in the central nervous system (CNS), tasked with the control of more sophisticated behaviors (70). However, the ENS and the CNS maintain intense crosstalk through reciprocal connections mediated by the VN (Figure 1) and pelvic nerve in mammals (71, 72). As the main substrate for this information exchange, the vagus nerve is an attractive target of neurostimulation therapies for the treatment of psychiatric and gastrointestinal disorders (73, 74).

The GI tract harbors a complex microbial ecosystem (Figure 1), consisting of bacteria, archaea, protists, and eukaryotic and prokaryotic viruses, also known as bacteriophages (75–77). The human microbiome has coevolved with its host (78), which keeps a tight leash on the intrinsic competitive nature of the microorganisms that comprise the microbiome, through both the nervous (71, 79, 80) and the immune systems (81, 82). This arrangement maximizes the benefits the host gains from the symbiotic relationship, including protection against pathogens, improved nutrition, and mental health (81). A sub-type of intestinal epithelial cells called enteroendocrine cells, provide a signaling pathway through which the microbiome interacts with the CNS via the vagus nerve (20, 83). Enteroendocrine cells have diverse phenotypes and express a variety of peptides/hormones that can act as signaling molecules on distinct targets, both local and distant, and some are chemoreceptors responding to a variety of luminal stimuli (84, 85). As other intestinal epithelial cells, enteroendocrine cells express toll-like receptors (86), allowing them to detect bacterial products, and activate vagal afferents through basal processes called neuropods (see Figure 1) (20, 87).

## The Gut Microbiome and Brain Function

There is increasing evidence of the association between microbiome dysfunction and CNS-related co-morbidities, such as anxiety, depression, autism spectrum disorders, Alzheimer's disease and PD (88–92). This association probably arose as a by-product of natural selection forces acting on microorganisms to adapt to the host and vice-versa (93). The effect of the microbiota on the CNS can lead to behavior modifications (93–95) and even to host manipulation (96) associated with increasing fitness of its bacterial populations. For instance, the microbiome can influence social interactions by acting on the nutritional behavior of individual animals, particularly those from social species where individuals share microbes and interact around foods (97). The proximate neuro-endocrinological and inflammatory mechanisms underlying this type of host manipulation are largely shared by the microbiome and the host (98, 99). For instance, levels of many neurotransmitters that are important for the expression of social behavior, such as serotonin (5-HT), dopamine, norepinephrine (NE), γ-aminobutyric acid (GABA), and glutamate are either expressed or regulated by bacteria (100–102). Particularly, most of the body's serotonin (5-HT) (5-hydroxytryptamine) is produced in the gut by enterochromaffin cells (EC) under the influence of the microbiome (103). The activation of 5-HT_4_ receptors induces the maturation of the ENS and regulates its adult function (104). In the gut, there are three major metabolic pathways leading from the essential amino acid tryptophan (Trp) to 5-HT, kynurenine (Kyn), and indole derivatives, which are under the direct or indirect control of the microbiota (105). During inflammatory states, most tryptophan is diverted to the production of Kyn and its metabolites kynurenine acid (KYNA) and quinolinic acid (QUIN) (106). While KYNA is considered neuroprotective, QUIN can cause excitotoxicity as an agonist of N-methyl-d-aspartate (NMDA) receptor and contribute to the neuropathogenesis of PD [for review, see (107)].

Although α-syn aggregates are also seen in the ENS of normally aging subjects (108), especially in the appendix (109), it is more prevalent in PD patients (110). Recent *in vivo* models showed that accumulation of α-syn aggregates in the ENS can be induced by alterations in the gut microbiome (111). Interestingly, Sampson et al. (112) demonstrated in mice, genetically modified to overexpress α-syn, that the presence of gut microbiota is necessary to promote pathological alterations and motor deficits similar to PD. They also demonstrated that fecal transplants from PD patients impair motor function in the same mouse strain, strongly suggesting that gut microbes may play a pivotal role in the onset of synucleinopathies such as PD (112). Underlying these findings is the fact that microbial amyloids produced by some members of the gut microbiota can be released in the extracellular space, where they can be internalized by neighboring cells, including neurons, and seed the formation of pathological aggregates of endogenous α-syn through permissive templating (113, 114). The failure of normal clearance mechanisms such as the ubiquitin-proteasome system, characteristic of both familial and idiopathic PD (115), to degrade the misfolded protein, may facilitate the seeding process.

The concept of microbial dysbiosis also comprises the bacteriophage components of the microbiome (116). Bacteriophages (phages) are viral parasites of bacteria and are important regulators of host-microbiome interactions through horizontal gene transfer and antagonistic coevolution (117, 118). Besides targeting bacteria, phages can impact human health by playing a direct role on intestinal inflammatory processes (119) and possibly causing α-syn misfolding (120). A recent study showed significant differences in the gut phagobiota of PD patients and healthy individuals and a depletion of *Lactococcus* bacteria (121) in the former, which is associated with the regulation of gut permeability (122) and dopamine production (102), two factors linked with the early signs of PD in the gut (123). Phage therapy has recently returned to the spotlight as an alternative antimicrobial strategy (124, 125). Eventually, it may also contribute to fighting PD through targeted approaches to manipulate the microbiome (121).

Probiotic bacteria have been linked to improved GI symptoms associated with PD (126). Probiotics affect the functionality of the CNS through beneficial interactions with the commensal gut microbiota and modulation of gut-derived inflammation (127). The microbiota of PD patients exhibits a pro-inflammatory profile (128, 129) due to increased intestinal permeability to endotoxins (lipopolysaccharide) (130). Bacterial amyloids may also favor a pro-inflammatory environment in the gut (131). A common bacterial component, the Curli fimbriae, share structural and biophysical properties with amyloids and are produced by *E. coli* through coordinated biosynthetic processes (132). Other components of the gut microbiome are also known to produce functional extracellular amyloids [e.g., *Salmonella, Klebsiella, Citrobacter*, and *Bacillus* species; (133)]. Since probiotic treatment induces an anti-inflammatory peripheral immune response in multiple sclerosis patients (134) there is a possibility they may also be beneficial for PD patients, although there are no reports corroborating this hypothesis. One option is to take advantage of Lactobacilli's ability to inhibit the formation of biofilms by pathogenic bacteria (135, 136). One caveat, however, is that the effects of probiotics are highly variable, being person-specific, as shown in a recent study (137). This limitation may be counteracted with the use of genetically-modified probiotics able to deliver novel therapeutics efficiently and with site specificity (138). Despite the increasing number of probiotic products available to consumers and the aggressive marketing proclaiming their efficacy, there have been few studies addressing concerns about efficacy and, more importantly, the safety of these products (139). There is an urgent need for more studies about the therapeutic potential of specific bacterial strains to help maintain oxidative and protein homeostasis in the ENS.

## Concluding Remarks

Aging is the main risk factor for the development of PD (140) and delaying the aging process is neuroprotective to PD in animal models (141). Aging is also associated with the accumulation of neuroinflammatory sequelae and the breakdown of homeostatic mechanisms that protect against protein misfolding, oxidative stress, decreased mitochondrial function, etc. The gut, as one of the main gateways to environmental exposure to the brain, may contribute to increasing the susceptibility to these factors. The microbiome has a protective effect mediating this exposure, and dysbiosis seems to be a pivotal risk factor for PD and other neurological disorders. Thus, the adoption of preventive measures to ensure a healthy microbiome throughout the lifetime can potentially decrease the risk of developing PD and other neurodegenerative diseases. The widespread use of antibiotics, for instance, which can kill gut bacteria indiscriminately, can cause a shift of the microbiome to an alternative stable state with unknown consequences in the long term (142).

## Author Contributions

All authors listed have made a substantial, direct and intellectual contribution to the work, and approved it for publication.

### Conflict of Interest Statement

The authors declare that the research was conducted in the absence of any commercial or financial relationships that could be construed as a potential conflict of interest.

## References

[1] Dauer W Przedborski S. Parkinson's disease: mechanisms and models. Neuron. (2003) 39:889–909. 10.1016/S0896-6273(03)00568-3 12971891

[2] Hughes AJ Daniel SE Kilford L Lees AJ. Accuracy of clinical diagnosis of idiopathic Parkinson's disease: a clinico-pathological study of 100 cases. J Neurol Neurosurg Psychiatry. (1992) 55:181–4. 1564476 10.1136/jnnp.55.3.181 PMC1014720

[3] Garcia-Ruiz PJ Chaudhuri KR Martinez-Martin P. Non-motor symptoms of Parkinson's disease A review…from the past. J Neurol Sci. (2014) 338:30–3. 10.1016/j.jns.2014.01.002 24433931

[4] Braak H Rüb U Gai WP Del Tredici K. Idiopathic Parkinson's disease: possible routes by which vulnerable neuronal types may be subject to neuroinvasion by an unknown pathogen. J Neural Transm. (2003) 110:517–36. 10.1007/s00702-002-0808-2 12721813

[5] Doty RL Deems DA Stellar S Olfactory dysfunction in parkinsonism A general deficit unrelated to neurologic signs, disease stage, or disease duration. Neurology. (1988) 38:1237–1237. 3399075 10.1212/wnl.38.8.1237

[6] Pearce RK Hawkes CH Daniel SE. The anterior olfactory nucleus in Parkinson's disease. Mov Disord. (1995) 10:283–7. 10.1002/mds.870100309 7651444

[7] Lewy F Paralysis agitans pathologische anatomie. In: Lewandowski M editor. Handbuch der Neurologie. Berlin: Springer (1912). p. 920–33.

[8] Okazaki H Lipkin LE Aronson SM. Diffuse intracytoplasmic ganglionic inclusions (Lewy type) associated with progressive dementia and quadriparesis in flexion. J Neuropathol Exp Neurol. (1961) 20:237–44. 13730588 10.1097/00005072-196104000-00007

[9] Polymeropoulos MH Lavedan C Leroy E Ide SE Dehejia A Dutra A. Mutation in the α-synuclein gene identified in families with Parkinson's disease. Science. (1997) 276:2045–7. 10.1126/science.276.5321.2045 9197268

[10] Gibb WR Lees AJ. The relevance of the Lewy body to the pathogenesis of idiopathic Parkinson's disease. J Neurol Neurosurg Psychiatry. (1988) 51:745–52. 10.1136/jnnp.51.6.745 2841426 PMC1033142

[11] Goedert M. Alpha-synuclein and neurodegenerative diseases. Nat Rev Neurosci. (2001) 2:492–501. 10.1038/35081564 11433374

[12] Gründemann J Schlaudraff F Haeckel O Liss B. Elevated α-synuclein mRNA levels in individual UV-laser-microdissected dopaminergic substantia nigra neurons in idiopathic Parkinson's disease. Nucleic Acids Res. (2008) 36:e38. 10.1093/nar/gkn084 18332041 PMC2367701

[13] Spillantini MG Crowther RA Jakes R Hasegawa M Goedert M. α-Synuclein in filamentous inclusions of Lewy bodies from Parkinson's disease and dementia with Lewy bodies. Proc Natl Acad Sci USA. (1998) 95:6469–73. 10.1073/pnas.95.11.6469 9600990 PMC27806

[14] Stefanis L. α-Synuclein in Parkinson's disease. Cold Spring Harb Perspect Med. (2012) 2:a009399. 10.1101/cshperspect.a009399 22355802 PMC3281589

[15] Uversky VN Dunker AK. Understanding protein non-folding. Biochim Biophys Acta BBA Proteins Proteomics. (2010) 1804:1231–64. 10.1016/j.bbapap.2010.01.017 20117254 PMC2882790

[16] Esteras-Chopo A Serrano L López de la Paz M. The amyloid stretch hypothesis: recruiting proteins toward the dark side. Proc Natl Acad Sci USA. (2005) 102:16672–7. 10.1073/pnas.0505905102 16263932 PMC1283810

[17] Uversky VN. Wrecked regulation of intrinsically disordered proteins in diseases: pathogenicity of deregulated regulators. Front Mol Biosci. (2014) 1:6. 10.3389/fmolb.2014.00006 25988147 PMC4428494

[18] Tuttle MD Comellas G Nieuwkoop AJ Covell DJ Berthold DA Kloepper KD. Solid-state NMR structure of a pathogenic fibril of full-length human α-synuclein. Nat Struct Mol Biol. (2016) 23:409–15. 10.1038/nsmb.3194 27018801 PMC5034296

[19] Rose AS Bradley AR Valasatava Y Duarte JM Prlic A Rose PW. NGL viewer: web-based molecular graphics for large complexes. Bioinforma. (2018) 34:3755–8. 10.1093/bioinformatics/bty419 29850778 PMC6198858

[20] Kaelberer MM Buchanan KL Klein ME Barth BB Montoya MM Shen X. A gut-brain neural circuit for nutrient sensory transduction. Science. (2018) 361:eaat5236. 10.1126/science.aat5236 30237325 PMC6417812

[21] Bohórquez DV Shahid RA Erdmann A Kreger AM Wang Y Calakos N. Neuroepithelial circuit formed by innervation of sensory enteroendocrine cells. J Clin Invest. (2015) 125:782–6. 10.1172/JCI78361 25555217 PMC4319442

[22] Anselmi L Bove C Coleman FH Le K Subramanian MP Venkiteswaran K. Ingestion of subthreshold doses of environmental toxins induces ascending Parkinsonism in the rat. NPJ Parkinson Dis. (2018) 4:30. 10.1038/s41531-018-0066-0 30302391 PMC6160447

[23] Hawkes CH Del Tredici K Braak H. Parkinson's disease: a dual-hit hypothesis. Neuropathol Appl Neurobiol. (2007) 33:599–614. 10.1111/j.1365-2990.2007.00874.x 17961138 PMC7194308

[24] Gershon MD Bursztajn S. Properties of the enteric nervous system: limitation of access of intravascular macromolecules to the myenteric plexus and muscularis externa. J Comp Neurol. (1978) 180:467–88. 10.1002/cne.901800305 659670

[25] Mellert TK Getchell ML Sparks L Getchell TV. Characterization of the immune barrier in human olfactory mucosa. Otolaryngol Head Neck Surg. (1992) 106:181–8. 1738551

[26] Attems J Walker L Jellinger KA. Olfaction and aging: a mini-review. Gerontology. (2015) 61:485–90. 10.1159/000381619 25968962

[27] Bowman GL Dayon L Kirkland R Wojcik J Peyratout G Severin IC. Blood-brain barrier breakdown, neuroinflammation, and cognitive decline in older adults. Alzheimer Dement. (2018) 14:1640–50. 10.1016/j.jalz.2018.06.2857 30120040

[28] Reeve A Simcox E Turnbull D. Ageing and Parkinson's disease: why is advancing age the biggest risk factor? Ageing Res Rev. (2014) 14:19–30. 10.1016/j.arr.2014.01.004 24503004 PMC3989046

[29] Okuzumi A Kurosawa M Hatano T Takanashi M Nojiri S Fukuhara T. Rapid dissemination of alpha-synuclein seeds through neural circuits in an in-vivo prion-like seeding experiment. Acta Neuropathol Commun. (2018) 6:96. 10.1186/s40478-018-0587-0 30231908 PMC6145187

[30] Steiner JA Quansah E Brundin P. The concept of alpha-synuclein as a prion-like protein: ten years after. Cell Tissue Res. (2018) 373:161–73. 10.1007/s00441-018-2814-1 29480459 PMC6541204

[31] Holmqvist S Chutna O Bousset L Aldrin-Kirk P Li W Björklund T. Direct evidence of Parkinson pathology spread from the gastrointestinal tract to the brain in rats. Acta Neuropathol. (2014) 128:805–20. 10.1007/s00401-014-1343-6 25296989

[32] Eisenberg D Jucker M. The amyloid state of proteins in human diseases. Cell. (2012) 148:1188–203. 10.1016/j.cell.2012.02.022 22424229 PMC3353745

[33] Stopschinski BE Diamond MI. The prion model for progression and diversity of neurodegenerative diseases. Lancet Neurol. (2017) 16:323–32. 10.1016/S1474-4422(17)30037-6 28238712

[34] Lawson VA Furness JB Klemm HM Pontell L Chan E Hill AF. The brain to gut pathway: a possible route of prion transmission. Gut. (2010) 59:1643–51. 10.1136/gut.2010.222620 21071583

[35] Ulusoy A Phillips RJ Helwig M Klinkenberg M Powley TL Di Monte DA. Brain-to-stomach transfer of α-synuclein via vagal preganglionic projections. Acta Neuropathol. (2017) 133:381–93. 10.1007/s00401-016-1661-y 28012041 PMC5326583

[36] Liu B Fang F Pedersen NL Tillander A Ludvigsson JF Ekbom A. Vagotomy and Parkinson disease: a Swedish register-based matched-cohort study. Neurology. (2017a) 88:1996–2002. 10.1212/WNL.0000000000003961 28446653 PMC5440238

[37] Svensson E Horváth-Puhó E Thomsen RW Djurhuus JC Pedersen L Borghammer P. Vagotomy and subsequent risk of Parkinson's disease. Ann Neurol. (2015) 78:522–9. 10.1002/ana.24448 26031848

[38] Kordower JH Chu Y Hauser RA Freeman TB Olanow CW. Lewy body-like pathology in long-term embryonic nigral transplants in Parkinson's disease. Nat Med. (2008a) 14:504–6. 10.1038/nm1747 18391962

[39] Kordower JH Chu Y Hauser RA Olanow CW Freeman TB. Transplanted dopaminergic neurons develop PD pathologic changes: a second case report. Mov Disord. (2008b) 23:2303–6. 10.1002/mds.22369 19006193

[40] Li J-Y Englund E Holton JL Soulet D Hagell P Lees AJ. Lewy bodies in grafted neurons in subjects with Parkinson's disease suggest host-to-graft disease propagation. Nat Med. (2008) 14:501–3. 10.1038/nm1746 18391963

[41] Recasens A Dehay B Bové J Carballo-Carbajal I Dovero S Pérez-Villalba A. Lewy body extracts from Parkinson disease brains trigger α-synuclein pathology and neurodegeneration in mice and monkeys. Ann Neurol. (2014) 75:351–62. 10.1002/ana.24066 24243558

[42] Jang H Boltz DA Webster RG Smeyne RJ. Viral parkinsonism. Biochim Biophys Acta. (2009) 1792:714–21. 10.1016/j.bbadis.2008.08.001 18760350 PMC4642437

[43] von Economo C Encephalitis lethargica: its sequelae and treatment. JAMA. (1931) 98:255 10.1001/jama.1932.02730290071039

[44] Langston JW. The MPTP story. J Parkinson Dis. (2017) 7:S11–S19. 10.3233/JPD-179006 28282815 PMC5345642

[45] Chiba K Trevor A Castagnoli N. Metabolism of the neurotoxic tertiary amine, MPTP, by brain monoamine oxidase. Biochem Biophys Res Commun. (1984) 120:574–8. 6428396 10.1016/0006-291x(84)91293-2

[46] Javitch JA D'Amato RJ Strittmatter SM Snyder SH. Parkinsonism-inducing neurotoxin, N-methyl-4-phenyl-1,2,3,6 -tetrahydropyridine: uptake of the metabolite N-methyl-4-phenylpyridine by dopamine neurons explains selective toxicity. Proc Natl Acad Sci USA. (1985) 82:2173–7. 3872460 10.1073/pnas.82.7.2173 PMC397515

[47] Villageliú DN Borts DJ Lyte M. Production of the neurotoxin salsolinol by a gut-associated bacterium and its modulation by alcohol. Front Microbiol. (2018) 9:3092. 10.3389/fmicb.2018.03092 30619171 PMC6305307

[48] Kurnik-Łucka M Panula P Bugajski A Gil K. Salsolinol: an unintelligible and double-faced molecule—lessons learned from *in vivo* and *in vitro* experiments. Neurotox Res. (2018) 33:485–514. 10.1007/s12640-017-9818-6 29063289 PMC5766726

[49] Thomas B Beal MF Parkinson's disease. Hum Mol Genet. (2007) 16:R183–94. 10.1093/hmg/ddm159 17911161

[50] Garcia-Ruiz PJ Espay AJ. Parkinson disease: an evolutionary perspective. Front Neurol. (2017) 8:157. 10.3389/fneur.2017.00157 28507529 PMC5410593

[51] Jomova K Vondrakova D Lawson M Valko M. Metals, oxidative stress and neurodegenerative disorders. Mol Cell Biochem. (2010) 345:91–104. 10.1007/s11010-010-0563-x 20730621

[52] Kalinderi K Bostantjopoulou S Fidani L. The genetic background of Parkinson's disease: current progress and future prospects. Acta Neurol Scand. (2016) 134:314–26. 10.1111/ane.12563 26869347

[53] Gluckman PD Hanson MA. Living with the past: evolution, development, and patterns of disease. Science. (2004) 305:1733–6. 10.1126/science.1095292 15375258

[54] Tooby J Cosmides L The past explains the present: emotional adaptations and the structure of ancestral environments. Ethol Sociobiol. (1990) 11:375–424.

[55] Murphy DD Rueter SM Trojanowski JQ Lee VM. Synucleins are developmentally expressed, and alpha-synuclein regulates the size of the presynaptic vesicular pool in primary hippocampal neurons. J Neurosci. (2000) 20:3214–20. 10.1523/jneurosci.20-09-03214.2000 10777786 PMC6773130

[56] Burré J Sharma M Tsetsenis T Buchman V Etherton MR Südhof TC. Alpha-synuclein promotes SNARE-complex assembly *in vivo* and *in vitro*. Science. (2010) 329:1663–7. 10.1126/science.1195227 20798282 PMC3235365

[57] Caspari R Lee S-H. Older age becomes common late in human evolution. Proc Natl Acad Sci USA. (2004) 101:10895–900. 10.1073/pnas.0402857101 15252198 PMC503716

[58] Kaushik S Cuervo AM. Proteostasis and aging. Nat Med. (2015) 21:1406–15. 10.1038/nm.4001 26646497

[59] Powers ET Morimoto RI Dillin A Kelly JW Balch WE. Biological and chemical approaches to diseases of proteostasis deficiency. Annu Rev Biochem. (2009) 78:959–91. 10.1146/annurev.biochem.052308.114844 19298183

[60] Balch WE Morimoto RI Dillin A Kelly JW. Adapting proteostasis for disease intervention. Science. (2008) 319:916–9. 10.1126/science.1141448 18276881

[61] Kikis EA Gidalevitz T Morimoto RI. Protein homeostasis in models of aging and age-related conformational disease. In: Tavernarakis N. editor. Protein Metabolism and Homeostasis in Aging Advances in Experimental Medicine and Biology. Boston, MA: Springer (2010). p. 138–59. 10.1007/978-1-4419-7002-2_11 PMC3402352 20886762

[62] Bobela W Aebischer P Schneider BL Alpha-synuclein as a mediator in the interplay between aging and Parkinson's disease. Biomolecules. (2015) 5:2675–700. 10.3390/biom5042675 26501339 PMC4693253

[63] Carabotti M Scirocco A Maselli MA Severi C. The gut-brain axis: interactions between enteric microbiota, central and enteric nervous systems. Ann Gastroenterol Q Publ Hell Soc Gastroenterol. (2015) 28:203–9. 25830558 PMC4367209

[64] Diaz Heijtz R Wang S Anuar F Qian Y Bjorkholm B Samuelsson A. Normal gut microbiota modulates brain development and behavior. Proc Natl Acad Sci USA. (2011) 108:3047–52. 10.1073/pnas.1010529108 21282636 PMC3041077

[65] Kelava I Rentzsch F Technau U. Evolution of eumetazoan nervous systems: insights from cnidarians. Philos Trans R Soc B Biol Sci. (2015) 370:20150065. 10.1098/rstb.2015.0065 26554048 PMC4650132

[66] Furness JB Stebbing MJ. The first brain: species comparisons and evolutionary implications for the enteric and central nervous systems. Neurogastroenterol Motil. (2018) 30:13234. 10.1111/nmo.13234 29024273

[67] Koizumi O Origin and evolution of the nervous system considered from the diffuse nervous system of cnidarians. In: Goffredo S Dubinsky Z editors. The Cnidaria, Past, Present and Future: The World of Medusa and Her Sisters. Cham: Springer International Publishing (2016). p. 73–91.

[68] Shimizu H Koizumi O Fujisawa T. Three digestive movements in Hydra regulated by the diffuse nerve net in the body column. J Comp Physiol A Neuroethol Sens Neural Behav Physiol. (2004) 190:623–30. 10.1007/s00359-004-0518-3 15168068

[69] Costa M Brookes SJH Hennig GW. Anatomy and physiology of the enteric nervous system. Gut. (2000) 47:iv15–9. 10.1136/gut.47.suppl_4.iv15 11076898 PMC1766806

[70] Kaas J Evolution of Nervous Systems. 2nd ed. Academic Press (2017). Available online at: https://www.sciencedirect.com/referencework/9780128040966/evolution-of-nervous-systems (accessed April 6, 2019).

[71] Powell N Walker MM Talley NJ. The mucosal immune system: master regulator of bidirectional gut-brain communications. Nat Rev Gastroenterol Hepatol. (2017) 14:143–59. 10.1038/nrgastro.2016.191 28096541

[72] Rao M Gershon MD. The bowel and beyond: the enteric nervous system in neurological disorders. Nat Rev Gastroenterol Hepatol. (2016) 13:517–28. 10.1038/nrgastro.2016.107 27435372 PMC5005185

[73] Breit S Kupferberg A Rogler G Hasler G. Vagus nerve as modulator of the brain–gut axis in psychiatric and inflammatory disorders. Front Psychiatry. (2018) 9:44. 10.3389/fpsyt.2018.00044 29593576 PMC5859128

[74] Johnson RL Wilson CG. A review of vagus nerve stimulation as a therapeutic intervention. J Inflamm Res. (2018) 11:203–13. 10.2147/JIR.S163248 29844694 PMC5961632

[75] Gerritsen J Smidt H Rijkers GT de Vos WM. Intestinal microbiota in human health and disease: the impact of probiotics. Genes Nutr. (2011) 6:209–40. 10.1007/s12263-011-0229-7 21617937 PMC3145058

[76] Ley RE Lozupone CA Hamady M Knight R Gordon JI. Worlds within worlds: evolution of the vertebrate gut microbiota. Nat Rev Microbiol. (2008) 6:776–88. 10.1038/nrmicro1978 18794915 PMC2664199

[77] Selber-Hnatiw S Rukundo B Ahmadi M Akoubi H Al-Bizri H Aliu AF. Human gut microbiota: toward an ecology of disease. Front Microbiol. (2017) 8:1265. 10.3389/fmicb.2017.01265 28769880 PMC5511848

[78] Moeller AH Caro-Quintero A Mjungu D Georgiev AV Lonsdorf EV Muller MN. Cospeciation of gut microbiota with hominids. Science. (2016) 353:380–2. 10.1126/science.aaf3951 27463672 PMC4995445

[79] Bailey MT Dowd SE Galley JD Hufnagle AR Allen RG Lyte M. Exposure to a social stressor alters the structure of the intestinal microbiota: implications for stressor-induced immunomodulation. Brain Behav Immun. (2011) 25:397–407. 10.1016/j.bbi.2010.10.023 21040780 PMC3039072

[80] Galley JD Nelson MC Yu Z Dowd SE Walter J Kumar PS. Exposure to a social stressor disrupts the community structure of the colonic mucosa-associated microbiota. BMC Microbiol. (2014) 14:189. 10.1186/1471-2180-14-189 25028050 PMC4105248

[81] Foster KR Schluter J Coyte KZ Rakoff-Nahoum S. The evolution of the host microbiome as an ecosystem on a leash. Nature. (2017) 548:43–51. 10.1038/nature23292 28770836 PMC5749636

[82] Zilber-Rosenberg I Rosenberg E. Role of microorganisms in the evolution of animals and plants: the hologenome theory of evolution. FEMS Microbiol Rev. (2008) 32:723–35. 10.1111/j.1574-6976.2008.00123.x 18549407

[83] Hoffman BU Lumpkin EA. A gut feeling. Science. (2018) 361:1203–4. 10.1126/science.aau9973 30237346

[84] Feher J 8.3—Intestinal and colonic chemoreception and motility. In: Feher J editor. Quantitative Human Physiology, 2nd Edn. Boston: Academic Press (2017). p. 796–809.

[85] Latorre R Sternini C De Giorgio R Greenwood-Van Meerveld B. Enteroendocrine cells: a review of their role in brain-gut communication. Neurogastroenterol Motil. (2016) 28:620–30. 10.1111/nmo.12754 26691223 PMC4842178

[86] Bogunovic M Davé SH Tilstra JS Chang DTW Harpaz N Xiong H. Enteroendocrine cells express functional Toll-like receptors. Am J Physiol Gastrointest Liver Physiol. (2007) 292:G1770–1783. 10.1152/ajpgi.00249.2006 17395901 PMC3203538

[87] Liddle RA. Neuropods. Cell Mol Gastroenterol Hepatol. (2019) 7:739–47. 10.1016/j.jcmgh.2019.01.006 30710726 PMC6463090

[88] Dinan TG Cryan JF Gut feelings on ParkinsonŠs and depression. Cerebrum. (2017) 2017:cer-04-17. PMC5501039 28698775

[89] Hsiao EY McBride SW Hsien S Sharon G Hyde ER McCue T. Microbiota modulate behavioral and physiological abnormalities associated with neurodevelopmental disorders. Cell. (2013) 155:1451–63. 10.1016/j.cell.2013.11.024 24315484 PMC3897394

[90] Kelly JR Minuto C Cryan JF Clarke G Dinan TG. Cross talk: the microbiota and neurodevelopmental disorders. Front Neurosci. (2017) 11:490. 10.3389/fnins.2017.00490 28966571 PMC5605633

[91] Pellegrini C Antonioli L Colucci R Blandizzi C Fornai M. Interplay among gut microbiota, intestinal mucosal barrier and enteric neuro-immune system: a common path to neurodegenerative diseases? Acta Neuropathol. (2018) 136:345–61. 10.1007/s00401-018-1856-5 29797112

[92] Unger MM Spiegel J Dillmann K-U Grundmann D Philippeit H Bürmann J. Short chain fatty acids and gut microbiota differ between patients with Parkinson's disease and age-matched controls. Parkinson Relat Disord. (2016) 32:66–72. 10.1016/j.parkreldis.2016.08.019 27591074

[93] Johnson KV-A Foster KR. Why does the microbiome affect behaviour? Nat Rev Microbiol. (2018) 16:647–55. 10.1038/s41579-018-0014-3 29691482

[94] Akami M Andongma AA Zhengzhong C Nan J Khaeso K Jurkevitch E. Intestinal bacteria modulate the foraging behavior of the oriental fruit fly Bactrocera dorsalis (Diptera: Tephritidae). PLoS ONE. (2019) 14:e0210109. 10.1371/journal.pone.0210109 30650116 PMC6334898

[95] Schretter CE Vielmetter J Bartos I Marka Z Marka S Argade S. A gut microbial factor modulates locomotor behaviour in *Drosophila*. Nature. (2018) 563:402. 10.1038/s41586-018-0634-9 30356215 PMC6237646

[96] Lewin-Epstein O Aharonov R Hadany L. Microbes can help explain the evolution of host altruism. Nat Commun. (2017) 8:14040. 10.1038/ncomms14040 28079112 PMC5241693

[97] Pasquaretta C Gómez-Moracho T Heeb P Lihoreau M. Exploring interactions between the gut microbiota and social behavior through nutrition. Genes. (2018) 9:E534. 10.3390/genes9110534 30404178 PMC6266758

[98] Freestone P. Communication between bacteria and their hosts. Scientifica. (2013) 2013:361073. 10.1155/2013/361073 24381789 PMC3871906

[99] Mazzoli R Pessione E. The Neuro-endocrinological Role of Microbial Glutamate and GABA Signaling. Front Microbiol. (2016) 7:1934. 10.3389/fmicb.2016.01934 27965654 PMC5127831

[100] Liu R Hong J Xu X Feng Q Zhang D Gu Y. Gut microbiome and serum metabolome alterations in obesity and after weight-loss intervention. Nat Med. (2017b) 23:859–68. 10.1038/nm.4358 28628112

[101] Strandwitz P Kim KH Terekhova D Liu JK Sharma A Levering J. GABA-modulating bacteria of the human gut microbiota. Nat Microbiol. (2019) 4:396. 10.1038/s41564-018-0307-3 30531975 PMC6384127

[102] Vodolazov IR Dbar SD Oleskin AV Stoyanova LG Exogenous and endogenous neuroactive biogenic amines: studies with *Lactococcus lactis* subsp. lactis Appl Biochem Microbiol. (2018) 54:603–10. 10.1134/S0003683818060157

[103] Yano JM Yu K Donaldson GP Shastri GG Ann P Ma L. Indigenous bacteria from the gut microbiota regulate host serotonin biosynthesis. Cell. (2015) 161:264–76. 10.1016/j.cell.2015.02.047 25860609 PMC4393509

[104] Vadder FD Grasset E Holm LM Karsenty G Macpherson AJ Olofsson LE. Gut microbiota regulates maturation of the adult enteric nervous system via enteric serotonin networks. Proc Natl Acad Sci USA. (2018) 115:6458–63. 10.1073/pnas.1720017115 29866843 PMC6016808

[105] Agus A Planchais J Sokol H. Gut microbiota regulation of tryptophan metabolism in health and disease. Cell Host Microbe. (2018) 23:716–24. 10.1016/j.chom.2018.05.003 29902437

[106] Keszthelyi D Troost FJ Jonkers DM van Donkelaar EL Dekker J Buurman WA. Does acute tryptophan depletion affect peripheral serotonin metabolism in the intestine? Am J Clin Nutr. (2012) 95:603–8. 10.3945/ajcn.111.028589 22301931

[107] Lim CK Fernández-Gomez FJ Braidy N Estrada C Costa C Costa S. Involvement of the kynurenine pathway in the pathogenesis of Parkinson's disease. Prog Neurobiol. (2017) 155:76–95. 10.1016/j.pneurobio.2015.12.009 27072742

[108] Böttner M Zorenkov D Hellwig I Barrenschee M Harde J Fricke T. Expression pattern and localization of alpha-synuclein in the human enteric nervous system. Neurobiol Dis. (2012) 48:474–80. 10.1016/j.nbd.2012.07.018 22850485

[109] Gray MT Munoz DG Gray DA Schlossmacher MG Woulfe JM. Alpha-synuclein in the appendiceal mucosa of neurologically intact subjects. Mov Disord. (2014) 29:991–8. 10.1002/mds.25779 24352892

[110] Barrenschee M Zorenkov D Böttner M Lange C Cossais F Scharf AB. Distinct pattern of enteric phospho-alpha-synuclein aggregates and gene expression profiles in patients with Parkinson's disease. Acta Neuropathol Commun. (2017) 5:1. 10.1186/s40478-016-0408-2 28057070 PMC5217296

[111] Chen SG Stribinskis V Rane MJ Demuth DR Gozal E Roberts AM. Exposure to the functional bacterial amyloid protein curli enhances alpha-synuclein aggregation in aged fischer 344 rats and *Caenorhabditis elegans*. Sci Rep. (2016) 6:34477. 10.1038/srep34477 27708338 PMC5052651

[112] Sampson TR Debelius JW Thron T Janssen S Shastri GG Ilhan ZE. Gut microbiota regulate motor deficits and neuroinflammation in a model of Parkinson's disease. Cell. (2016) 167:1469–80.e12. 10.1016/j.cell.2016.11.018 27912057 PMC5718049

[113] Friedland RP Chapman MR. The role of microbial amyloid in neurodegeneration. PLoS Pathog. (2017) 13:e1006654. 10.1371/journal.ppat.1006654 29267402 PMC5739464

[114] Soto C Pritzkow S. Protein misfolding, aggregation, and conformational strains in neurodegenerative diseases. Nat Neurosci. (2018) 21:1332–40. 10.1038/s41593-018-0235-9 30250260 PMC6432913

[115] McNaught KSP Olanow CW Halliwell B Isacson O Jenner P. Failure of the ubiquitin–proteasome system in Parkinson's disease. Nat Rev Neurosci. (2001) 2:589–94. 10.1038/35086067 11484002

[116] Manrique P Dills M Young MJ. The human gut phage community and its implications for health and disease. Viruses. (2017) 9:E141. 10.3390/v9060141 28594392 PMC5490818

[117] Duerkop BA. Bacteriophages shift the focus of the mammalian microbiota. PLoS Pathog. (2018) 14:e1007310. 10.1371/journal.ppat.1007310 30359456 PMC6201943

[118] Scanlan PD. Bacteria–bacteriophage coevolution in the human gut: implications for microbial diversity and functionality. Trends Microbiol. (2017) 25:614–23. 10.1016/j.tim.2017.02.012 28342597

[119] Gogokhia L Buhrke K Bell R Hoffman B Brown DG Hanke-Gogokhia C. Expansion of bacteriophages is linked to aggravated intestinal inflammation and colitis. Cell Host Microbe. (2019) 25:285–99.e8. 10.1016/j.chom.2019.01.008 30763538 PMC6885004

[120] Tetz G Tetz V Tetz G Tetz V. Bacteriophages as new human viral pathogens. Microorganisms. (2018b) 6:54. 10.3390/microorganisms6020054 29914145 PMC6027513

[121] Tetz G Brown SM Hao Y Tetz V. Parkinson's disease and bacteriophages as its overlooked contributors. Sci Rep. (2018a) 8:10812. 10.1038/s41598-018-29173-4 30018338 PMC6050259

[122] Darby TM Owens JA Saeedi BJ Luo L Matthews JD Robinson BS. *Lactococcus lactis* subsp. cremoris is an efficacious beneficial bacterium that limits tissue injury in the intestine iScience. (2019) 12:356–67. 10.1016/j.isci.2019.01.030 30739017 PMC6369221

[123] Houser MC Tansey MG. The gut-brain axis: is intestinal inflammation a silent driver of Parkinson's disease pathogenesis? NPJ Parkinson Dis. (2017) 3:3. 10.1038/s41531-016-0002-0 28649603 PMC5445611

[124] Chan BK Abedon ST Loc-Carrillo C. Phage cocktails and the future of phage therapy. Future Microbiol. (2013) 8:769–83. 10.2217/fmb.13.47 23701332

[125] Górski A Miedzybrodzki R Weber-Dabrowska B Fortuna W Letkiewicz S Rogóz P. Phage therapy: combating infections with potential for evolving from merely a treatment for complications to targeting diseases. Front Microbiol. (2016) 7:1515. 10.3389/fmicb.2016.01515 27725811 PMC5035766

[126] Barichella M Pacchetti C Bolliri C Cassani E Iorio L Pusani C. Probiotics and prebiotic fiber for constipation associated with Parkinson disease: an RCT. Neurology. (2016) 87:1274–80. 10.1212/WNL.0000000000003127 27543643

[127] Wang H Lee I-S Braun C Enck P. Effect of probiotics on central nervous system functions in animals and humans: a systematic review. J Neurogastroenterol Motil. (2016) 22:589–605. 10.5056/jnm16018 27413138 PMC5056568

[128] Bedarf JR Hildebrand F Coelho LP Sunagawa S Bahram M Goeser F Functional implications of microbial and viral gut metagenome changes in early stage L-DOPA-naïve Parkinson's disease patients. Genome Med. (2017) 9:39 10.1186/s13073-017-0428-y 28449715 PMC5408370

[129] Petrov VA Saltykova IV Zhukova IA Alifirova VM Zhukova NG Dorofeeva YB. Analysis of gut microbiota in patients with Parkinson's disease. Bull Exp Biol Med. (2017) 162:734–7. 10.1007/s10517-017-3700-7 28429209

[130] Forsyth CB Shannon KM Kordower JH Voigt RM Shaikh M Jaglin JA. Increased intestinal permeability correlates with sigmoid mucosa alpha-synuclein staining and endotoxin exposure markers in early Parkinson's disease. PLoS ONE. (2011) 6:e28032. 10.1371/journal.pone.0028032 22145021 PMC3228722

[131] Miraglia F Colla E. Microbiome, Parkinson's disease and molecular mimicry. Cells. (2019) 8:222. 10.3390/cells8030222 30866550 PMC6468760

[132] Taglialegna A Lasa I Valle J. Amyloid Structures as Biofilm Matrix Scaffolds. J Bacteriol. (2016) 198:2579–88. 10.1128/JB.00122-16 27185827 PMC5019065

[133] Van Gerven N Van der Verren SE Reiter DM Remaut H. The role of functional amyloids in bacterial virulence. J Mol Biol. (2018) 430:3657–84. 10.1016/j.jmb.2018.07.010 30009771 PMC6173799

[134] Tankou SK Regev K Healy BC Tjon E Laghi L Cox LM. A probiotic modulates the microbiome and immunity in multiple sclerosis. Ann Neurol. (2018) 83:1147–61. 10.1002/ana.25244 29679417 PMC6181139

[135] Söderling EM Marttinen AM Haukioja AL. Probiotic lactobacilli interfere with Streptococcus mutans biofilm formation *in vitro*. Curr Microbiol. (2011) 62:618–22. 10.1007/s00284-010-9752-9 20835828

[136] Vuotto C Longo F Donelli G. Probiotics to counteract biofilm-associated infections: promising and conflicting data. Int J Oral Sci. (2014) 6:189–94. 10.1038/ijos.2014.52 25257882 PMC5153589

[137] Zmora N Zilberman-Schapira G Suez J Mor U Dori-Bachash M Bashiardes S. Personalized gut mucosal colonization resistance to empiric probiotics is associated with unique host and microbiome features. Cell. (2018) 174:1388–405. 10.1016/j.cell.2018.08.041 30193112

[138] Singh B Mal G Marotta F. Designer probiotics: paving the way to living therapeutics. Trends Biotechnol. (2017) 35:679–82. 10.1016/j.tibtech.2017.04.001 28483159

[139] Cohen PA. Probiotic safety—no guarantees. JAMA Intern Med. (2018) 178:1577–8. 10.1001/jamainternmed.2018.5403 30242393

[140] Driver JA Logroscino G Gaziano JM Kurth T. Incidence and remaining lifetime risk of Parkinson disease in advanced age. Neurology. (2009) 72:432–8. 10.1212/01.wnl.0000341769.50075.bb 19188574 PMC2676726

[141] Cooper JF Dues DJ Spielbauer KK Machiela E Senchuk MM Raamsdonk JMV. Delaying aging is neuroprotective in Parkinson's disease: a genetic analysis in C. elegans models NPJ Parkinson Dis. (2015) 1:15022. 10.1038/npjparkd.2015.22 28725688 PMC5516561

[142] Dethlefsen L Relman DA. Incomplete recovery and individualized responses of the human distal gut microbiota to repeated antibiotic perturbation. Proc Natl Acad Sci USA. (2011) 108 (Suppl. 1):4554–61. 10.1073/pnas.1000087107 20847294 PMC3063582

